# Geophagy practices and the content of chemical elements in the soil eaten by pregnant women in artisanal and small scale gold mining communities in Tanzania

**DOI:** 10.1186/1471-2393-14-144

**Published:** 2014-04-15

**Authors:** Elias C Nyanza, Mary Joseph, Shahirose S Premji, Deborah SK Thomas, Cynthia Mannion

**Affiliations:** 1 School of Public Health, Catholic University of Health and Allied Sciences, P.O. Box 1464, Bugando Area, Mwanza, Tanzania; 2 Goodneighbours Tanzania, P.O. Box 33104, Dar es salaam, Boko Area, Kinondoni, Tanzania; 3 Faculty of Nursing, University of Calgary, University of Calgary, 2500 University Drive, NW, Calgary, AB T2N 1 N4, Canada; 4 Department of Community Health Sciences, Faculty of Medicine, University of Calgary, 3280 Hospital Drive NW, Calgary, AB T2N 4Z6, Canada; 5 Department of Geography & Environmental Sciences, University of Colorado Denver, PO Box 173364, Denver, CO 80217-3364, USA

**Keywords:** Geophagy, Pica, Pregnancy, Chemical elements, Soil pollution, Arsenic, Mercury

## Abstract

**Background:**

Geophagy, a form of pica, is the deliberate consumption of soil and is relatively common across Sub-Saharan Africa. In Tanzania, pregnant women commonly eat soil sticks sold in the market (*pemba*), soil from walls of houses, termite mounds, and ground soil (*kichuguu*). The present study examined geophagy practices of pregnant women in a gold mining area of Geita District in northwestern Tanzania, and also examined the potential for exposure to chemical elements by testing soil samples.

**Method:**

We conducted a cross sectional study using a convenience sample of 340 pregnant women, ranging in age from 15–49 years, who attended six government antenatal clinics in the Geita District, Tanzania. Structured interviews were conducted in June-August, 2012, to understand geophagy practices. In addition, soil samples taken from sources identified by pregnant women practicing geophagy were analysed for mineral element content.

**Results:**

Geophagy was reported by 155 (45.6%) pregnant women with 85 (54.8%) initiating the practice in the first trimester. A total of 101 (65%) pregnant women reported eating soil 2 to 3 times per day while 20 (13%) ate soil more than 3 times per day. Of 155 pregnant women 107 (69%) bought *pemba* from local shops, while 48 (31%) consumed ground soil *kichuguu*. The estimated mean quantity of soil consumed from *pemba* was 62.5 *grams/day*. Arsenic, chromium, copper, iron, manganese, nickel and zinc levels were found in both *pemba* and *kichuguu* samples*.* Cadmium and mercury were found only in the *kichuguu* samples. Based on daily intake estimates, arsenic, copper and manganese for *kichuguu* and copper and manganese for *pemb*a samples exceed the oral Minimum Risk Levels designated by the U.S. Agency for Toxic Substance and Disease Registry.

**Conclusion:**

Almost 50% of participants practiced geophagy in Geita District consistent with other reports from Africa. Both *pemba* and *kichuguu* contained chemical elements at varying concentration, mostly above MRLs. As such, pregnant women who eat soil in Geita District are exposed to potentially high levels of chemical elements, depending upon frequency of consumption, daily amount consumed and the source location of soil eaten.

## Background

Geophagy, the deliberate consumption of soil, is prevalent among pregnant women across Sub-Saharan African countries, such as Kenya, Ghana, Rwanda, Nigeria, Tanzania, and South Africa [1-9]. The prevalence of geophagy varies between and within countries, but is estimated between 10-75% [3-5,7]. It is likely that underreporting of geophagy occurs, for a variety of reasons, including embarrassment regarding the behavior, lack of knowledge and sensitive questioning on the part of investigator inquiring about geophagy and differing perceptions, beliefs, and cultural norms [4,10].

The etiology of geophagy remains elusive. Both physiologic (e.g., mineral deficiency or hunger) and psychological (e.g., craving, obsessive-compulsive spectrum disorder) models have been proposed [9-11]. Cultural and socioeconomic factors have also been identified as influencing the practice of geophagy, thereby highlighting its complex and little understood nature [10].

The health impacts of geophagy remain controversial and inconclusive, as reports in the literature show health benefits, harmful effects, and the absence of effects [1-3,10,12-15]. Substances with clay constituents have long been used (e.g., Kaopectate®) for treating gastroenteritis, nausea, diarrhea and vomiting [3,14,16]. Helminthes infection that leads to anemia due to blood loss from the intestine can result from geophagy. For example, a cohort study involving 108 pregnant women conducted in Ashanti region of Ghana, reported 54.9% with anemic cases and 17.6% with helminthes infections, of which geophagy, among other factors, was said to be a predisposing factor [17]. In contrast, studies have indicated that geophagy did not increase the risk of helminthes infection, but microbial content was high [2,18].

Soil consumed by pregnant women contains substances that are micronutrients and toxins [1]. Micronutrients include copper, iron, manganese, zinc and chromium, and are considered essential nutrients for humans [19-24]. Arsenic and lead are known toxins to humans and, depending upon exposure, have detrimental effects on human health if ingested. Other constituents commonly found in soil, such as cadmium and nickel, do not have sufficient evidence to support health benefits, but are known to be hazardous to humans given repeated doses over time [25]. Of particular concern is soil contaminated by human activities, such as mining, as this can increase exposure to environmental toxins if ingested.

The risk associated with the ingestion of contaminated soil depends on the element of interest, how much is consumed (dose), how often (frequency) and the bioavailability [26]. Bioavailability is broadly defined as the dose of an unchanged substance that is absorbed and consequently distributed throughout the body [26]. This can depend upon the form or state of a chemical element. Minerals, such as copper, iron, manganese, and zinc, can be in elemental, ionic, or chelated forms or in a colloid, all of which affect the rate of absorption. Some are changed by the contents of the gut, for example, if a meal has been consumed. Meal components can interact with minerals and increase, decrease or delay absorption. Nutrients can also interact with each other for example calcium which decreases iron and zinc absorption [27].

Some chemical elements may affect the gut prior to absorption. Iron is known to irritate the gut lining causing gastrointestinal distress, such as cramping and constipation [27]. Iron containing soil may contribute to gut irritation but not necessarily to increased iron absorption as that is regulated by iron metabolites in the body. Iron overload occurs mainly from hereditary conditions or long term intake of iron rich foods or supplements [27].

Arsenic, mercury, nickel and lead are sometimes referred to as toxic elements and have been linked to adverse reproductive outcomes, neurological disorders, and impaired cognitive development in children [28-36]. For example, results of a study done in Bangladesh suggested that maternal arsenic exposure early in pregnancy was associated with low birth weight [36]. Impaired cognitive function has been reported in children even with arsenic concentration in the urine below the established safe limit of 50 *μg/L*[36]. Maternal exposure from these toxins can concentrate in the fetus given its small size relative to the mother and the inability of the immature liver to detoxify blood. Evidence suggests that even low levels of trace metal exposure, such as cadmium and lead, are linked to numerous negative health outcomes, including cognitive deficits and other delayed developmental milestones [34,35,37].

In Tanzania, pregnant women commonly eat soil sticks sold daily in the market (called *pemba* in Swahili), soil from walls of houses, termite mounds, and ground soil (called *kichuguu* in Swahili). Tanzania has Africa’s second largest number of people engaged in artisanal and small-scale gold mining activities. The Geita Region, located on the shores of Lake Victoria, Tanzania, is comprised of five districts and has experienced continued significant growth in artisanal and gold mining [38]. Geita District (7,825 *km*^2^), with a total population of more than 807,617 (407,144 being female) [39], has several active artisanal gold mining communities along with large scale gold mining operations.

In 2011, a study carried out in one artisanal gold mine with minimal waste management practices in Geita [40], reported high levels of arsenic and mercury, among other chemical elements in the ground soil. Despite the risk from contaminated soil, the practice of geophagy in the Geita District remains undocumented. This study describes pregnant women’s soil eating practices and awareness of potential risks in communities surrounding mining areas in Geita District and examines the potential for exposure to chemical elements.

## Methods

### Study design

We conducted a cross-sectional study using structured interviews to document pregnant women’s soil eating practices and to understand their attitudes and beliefs about geophagy. Additionally, soil sampling was undertaken from the various sources of soil consumed by the pregnant women, which were tested for the presences of 10 chemical elements. *Kichuguu* was obtained from sites identified by the women participating in the study who answered affirmatively that they practiced geophagy, whereas *pemba* was obtained in local shops using convenience sampling.

### Setting

According to the “Annual 2011 Reproductive and Child Health (RCH) Report,” Geita District has an average of 53,803 pregnant women per year [41]. There are 53 government antenatal clinics serving the area that have the ability to receive up to 50 pregnant women per day per clinic [41]. The clinics provide reproductive and child health services, including Prevention of Mother-to-Child Transmission of HIV (PMTCT), family planning, birth preparedness planning, as well as focused antenatal care that includes checking blood pressure and body weight, provision of intermittent presumptive treatment for malaria, deworming and nutrient supplements such as folic acid and iron.

### Structured interviews

A convenience sample of 340 women consented to participate in face-to-face structured interviews from June 8, through July 30, 2012. Participants were from six villages; Geita (n = 165, 48.5%), Katolo (n = 652012, 19.1%), Rwamagasa (n = 40, 11.8%), Bukoli (n = 35, 10.3%), Kasamwa (n = 25, 7.4%) and Chikobe (n = 10, 2.9%).

All pregnant women attending the antenatal clinic who were 15 to 49 years, fluent in Swahili, and were not in distress (experiencing pain or discomfort, or demonstrating signs and symptoms of malaria) were considered eligible to participate. Young pregnant women are considered a mature minor in Tanzania, and so 15 years of age was the age lower limit of those recruited. Where the numbers of women attending the antenatal clinics were low (i.e., approximately 30 per day), all pregnant women were invited to participate; however, where the numbers were high (i.e., more than 30 or so per day), a systematic selection was employed whereby every third pregnant woman was invited to participate to limit selection bias. All women who were invited to participate in the study accepted and none withdrew from the study once enrolled.

**Figure 1:**
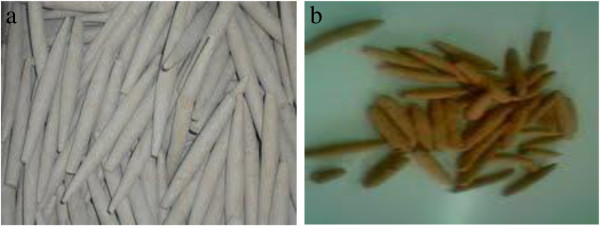
Pemba samples from (a) Musoma and (b) Kigoma; the difference in color is attributable to the increased levels of iron in those from Kigoma.

The interview questionnaire was translated to Swahili by the principal investigator and then back translated to English by a colleague to ensure language equivalency. The questionnaire was pilot tested with 20 pregnant women in one of the antenatal clinics in Mwanza in a nearby district, and subsequently revised. Pregnant women who reported practicing geophagy during pregnancy were also asked to identify their sources of soil. Some pregnant women were willing to show the researcher the exact location of the soil source so that a sample could be obtained. A total of fourteen (n = 14) samples were obtained from different ground sites all within Geita District, mostly termite mounds and a few from house mud walls (treated as *kichuguu* for this analysis)*.* Using a convenience sample strategy, eight (n = 8) *pemba* samples were obtained from the local market places, four (n = 4) originating from Musoma (northwestern, Tanzania, near Lake Victoria) and four (n = 4) originating from Kigoma (western, Tanzania, near Lake Tanganyika) (Figure 1).

### Structured interview analysis

Interview data were analysed using Statistical Package for Social Sciences (SPSS) version 17.0. Frequencies and percentages were reported for categorical and ordinal level data. Descriptive statistics were used to describe socio-demographic characteristics of pregnant women. Comparisons were made between women who indicated that they ate soil and those who indicated that they did not eat soil. Statements were categorized as “agree, uncertain, or disagree”. We also tested for differences across these categories as uncertainty influences decision-making and require an understanding of risk attitudes. Pearson’s Chi-square test or Fischer’s exact test (when expected cell counts were less than 5) was used when comparing categorical data. A p-value of less than .05 was considered statistically significant. We reported 95% confidence intervals. Verbal responses to open ended questions were reviewed and a codebook developed. Key words or phrases were independently coded and evaluated manually by two people in order to derive themes.

### Laboratory procedures

*Kichuguu* samples were air/sun dried, pounded, homogenized, and subsequently packed in a re-sealable plastic bag. *Pemba* were purchased from the shop and packed in a re-sealable plastic bag. Analyses were carried out at an International Standards Organization accredited laboratory (*ISO/IEC 17025:2005*) in Tanzania. All samples were sieved to less than 2 *mm* prior to acid digestion. For arsenic, cadmium, chromium, copper, iron, manganese, nickel, lead and zinc a weight of 2(±0.01) *grams* for each of the sieved soil samples were weighed using an analytical balance capable of recording up to three decimal place followed by the addition of 2.5(±0.1) *ml* concentrated Nitric acid (HNO_3)_ and later 2.5(±0.1) *ml* concentrated Hydrochloric acid (HCl). This was then digested at 110(±2)°C for 40 minutes followed by cooling and then the addition of 10 *ml* of 18.2*Ωm* de-ionized water. This was further digested for 20 minutes. The volume was increased to 50(±0.50) *ml* with 18.2*Ωm* de-ionized water and filtered through a 0.45 *μm* membrane filter and analysed using Inductively Coupled Plasma Mass Spectrometry (ICP-MS) [1,42,43]. For arsenic, 5(±0.03) *ml* of concentrated HCl was added to 15(±0.03) *ml* of the digest followed by an addition of 0.2(±0.02) *grams* of potassium iodide. This was analysed with the Hydride Generation Atomic Absorption Spectrophotometer (HGAAS) technique using 0.30% Sodium Borohydrate (NaBH_4_) and 0.25% Sodium Hydroxide (NaOH) as reductant [43,44].

Determination of total mercury used 1(±0.02) *grams* of the less than 2 *mm* sieved sample followed by the addition of 10(±0.05) *ml* of 18.2Ωm de-ionized water, 2 (±0.05) *ml* of concentrated Sulphuric acid (H_2_SO_4_) and 1(±0.05) *ml* concentration HNO_3_ with intermittent mixing between each addition. This was followed by an addition of 10(±0.05) *ml* 5%^*w*^*/*_*v*_ potassium permanganate and 2(±0.05) *ml* 5%^*w*^*/*_*v*_ potassium persulphate and digested at 95°C for 30 minutes. This was then followed by an addition of 5(±0.05) *ml* of hydroxylamine hydrochloride (10% ^*w*^*/*_*v*_) - sodium chloride (12% ^*w*^*/*_*v*_) solution to reduce excess potassium permanganate after cooling. The digest was increased to 50(±0.50) *ml* with 18.2Ωm de-ionized water [43,45,46]. Total mercury was determined by Cold Vapor Atomic Absorption Spectrophotometry (CVAAS) using 25% Tin (II) Chloride as reductant, as documented in the American Public Health Association Standard Methods [43,44,47] within 24 hours.

### Exposure estimate calculations

The soil ingestion rate *(Ig/R)* (*gram/day*) was estimated according to the basic equation documented in the UNEP Basic Environmental Health Handbook (*Ig/R = FNW)*[48]; where F; frequency of *pemba* eaten per day, N; number of *pemba* eaten at one time, W; mean weight of *pemba* (*grams*). The Daily Intake (DI) for a specific chemical element was estimated using the soil ingestion rate (*Ig/R*) of 62.5 *g/day* (the estimated amount of *pemba* eaten on average by women in the study) and the concentration of the particular chemical element (*DI* = *Ig/R x concentration of the chemical element)*[48].

The daily intake was converted to a dose (*mg/kg/day*), using a mean weight 80 *kg* for an adult of 21 or more years [46] because we did not have actual weights for our study participants. These were then compared to the oral Minimal Risk Levels (MRLs)^a^ established by the US Agency of Toxic Substances and Disease Registry (ATSDR) [26,45]. Chemical elements with a dose (*mg/kg/day*) less than the oral MRLs for intermediate (15 to 364 days) or chronic (≥365 days) exposures [45] were considered normal levels. We have also used the Dietary Reference Intakes (DRIs) developed by the Institute of Medicine as nutrient reference points to discuss risk levels of micronutrients found in soil, although we are aware that they are not intended for non-food substances. Tolerable upper intake levels (UL) were established for many micronutrients by the Institute of Medicine [25], which when consumed in amounts reaching or exceeding the UL can cause adverse effects.

### Ethical considerations

Ethical approval was obtained from Catholic University of Health and Allied Sciences and Bugando Medical Centre joint Research Ethical Committee. Permission to conduct research in Geita District was obtained from the respective authorities at the regional, district and village levels. Pregnant women were asked individually if they were interested in participating and then written informed consent was obtained.

## Results

### Geophagy practice, belief and perception among pregnant women

One third of the mothers enrolled (31.2%, n = 106) were between 21 to 26 years, 25% (n = 85) were between 15–20 years, 24.1% (n = 82) were between 27–32 years, 15.3% (n = 52) were between 33–40 years, and a few (4.4%, n = 15) were aged above 40 years. More than half (55.9%, n = 190) completed primary school, 34.7% (n = 118) had no formal education, and 9.4% (n = 32) had secondary education and above. One third (36.5%, n = 124) of the respondents were housewives, while 30.9% (105) were engaged in agriculture, including livestock keeping and cultivation and 15.9% (n = 54) were engaged in mining activities. Some of the respondents (13.5%, n = 46) were involved in business, which included all types of shops, such as tailoring, etc. A small number 3.2% (n = 11) were employed in public services.

Geophagy was practiced by 45.6% (n = 155) of these pregnant women enrolled. Reasons given for eating soil included a persistent desire (60.6%, n = 94), a need to reduce morning sickness (31%, n = 48), attraction by the scent of the soil (5.81%, n = 9), and enjoyment of the soil’s taste (2.6%, n = 4). Among the respondents who reported eating soil, most of them (65.2%, n = 101) ate soil 2–3 times a day, 21.3% (n = 33) ate soil once a day and 13.5% (n = 21) ate soil more than three times a day. Some of the respondents (31%, n = 48) who practiced geophagy consume *kichuguu*, but the majority (69%, n = 107) purchased *pemba* from the local shop/market.

Respondents reported initiating geophagy at various times during pregnancy; in the first trimester (i.e., 1st to 3^rd^ month; 54.8%, n = 85), in the second trimester (i.e., 4^th^ to 6^th^ month; 36.1%, n = 56), and in the third trimester (i.e., 7^th^ to 9^th^ month; 9%, n = 14). One quarter of participants (24.5%, n = 38/155) attempted to stop eating soil while the rest (75.5%, n = 117) indicated a persistent desire to eat soil because of the “good smell” of the soil and the need to stop vomiting.

Table 1 summarizes the identification of soil as a non-food substance by participants. More than half (59.7%, n = 203) of the 340 respondents identified soil as a substance that pregnant women consume, but not a “normal” food. Other substances consumed included charcoal (13.2%, n = 45), uncooked rice (1.8%, n = 6) and ice (0.88%, n = 3). A majority of the pregnant women (67.4%, n = 229) indicated that soil does not provide nutrients to mother or unborn baby, while only a few (3.2%, n = 11) indicated that soil provides nutrients to mother and unborn baby. Some of the respondents (29.4%, n = 100) were not sure whether eating soil provides nutrients to mother and unborn baby.

**Table 1:** Substances eaten by pregnant women which are not typically food

**Mentioned substances**	**N**	**%**
Soil	203	59.7
Charcoal	45	13.2
Uncooked rice	6	1.8
Ice	3	0.88
None*	83	24.4

*Unable to identify substances.

There was a statistically significant difference in beliefs between those who practiced and those who did not practice geophagy summarized in Table 2. For instance, more than half (58.7%, n = 91) of the pregnant women who practiced geophagy believed that eating soil stops/prevents morning sickness. However, a majority of the pregnant women (61.1, n = 113) who do not practice geophagy were uncertain (p < .001). More than half of those in the study practicing geophagy (57.4%, n = 89) did not believe that eating soil ensures healthy pregnancy, while a majority of those not practicing geophagy (54.1%, n = 100) were uncertain (p = .009). Likewise, pregnant women were uncertain whether eating soil is a sign of a woman being pregnant (p = .001) or ensures a beautiful baby (p = .021).

**Table 2:** Geophagy beliefs and practice *pearson chi-square

		**Geophagy practice**				
		**Yes**			**No**	
**Geophagy beliefs**		**n**	**%**	**N**	**%**	**p-value**
Eating soil reduces/stops	Agree	91	58.7	34	18.4	
morning sickness	Uncertain	48	31.0	113	61.1	< .001^*^
	Disagree	16	10.3	38	20.5	
Eating soil ensures	Agree	-	-	4	2.2	
healthy pregnancy	Uncertain	66	42.6	100	54.1	.009^^^
	Disagree	89	57.4	81	43.8	
Eating soil prevents	Agree	-	-	-	-	
prolonged labor	Uncertain	31	20.0	76	40.5	< .001^*^
	Disagree	124	80.0	109	58.9	
Eating soil is a sign of	Agree	11	7.1	16	8.6	
a woman being pregnant	Uncertain	44	28.4	87	47.0	.001^*^
	Disagree	100	64.5	82	44.3	
Eating soil ensures	Agree	1	0.60	2	1.1	
a beautiful baby	Uncertain	53	34.2	88	47.6	.021^^^
	Disagree	101	65.2	95	51.4	

*Pearson Chi-Square.

^Fisher’s Exact Test.

### Chemical elements in *pemba* and *kichuguu*

It was not possible to estimate the quantity of *kichuguu* eaten by pregnant women because they could not recall the amount eaten each time. However, it was possible to do so for *pemba* as respondents could indicate the number of sticks eaten each time and how many times per day. The total weight of soil eaten per day was estimated using mean weight of a *pemba* stick. Samples of *pemba* were taken to an ISO 17025 accredited laboratory where they were weighed. The mean weight of the *pemba* was 9.74 *grams*. Using this weight, it was determined that over half of the pregnant women who ate *pemba* (52.3%, n = 56) ate more than 50 *g/day*, 24.3% (n = 26) ate 20 to 50 *g/day*, and 21.5% (n = 23) ate less than 20 *g/day*. As such, the mean daily consumption was estimated to be 62.5 *g/day* for a pregnant woman for both *pemba* and *kichuguu*.

The concentration of chemical elements in both *pemba* and *kichuguu* are presented in Table 3. The concentration of chemical elements in *pemba* varied depending on the location sourced. Kigoma sourced *pemba* were high in chromium, copper, iron, nickel and zinc, while *pemba* from Musoma were high in manganese and lead. Mercury and cadmium were below the method detection limits for *pemba* from both sources. For *Kichuguu* samples, mercury and cadmium ranged from 0.015 to 0.075 *mg/kg* and <0.001 to 0.220 *mg/kg* respectively. Similar to *pemba*, chemical elements in the *kichuguu* samples varied from one area to another. However, the concentrations were higher overall for arsenic, cadmium, chromium, copper, mercury, nickel, lead and zinc as compared to the *pemba* samples. The concentrations of iron in the *kichuguu* samples were relatively low compared to the Kigoma *pemba* samples. The mean chemical element concentrations were used to estimate the Daily Intake (DI) and the dose for pregnant women in Geita District. Table 4 summarizes the estimated DI and daily dose of chemical elements for pregnant women consuming *pemba* and *kichuguu* samples.

**Table 3:** **Chemical element content in *pemba *** **and *kichuguu *** **eaten by pregnant women**

	**Location**	***Sample***	**Total chemical element in *mg/kg***									
	**Sourced**	***Identity***	***As***	***Cd***	***Cr***	***Cu***	***Fe***	***Hg***	***Mn***	***Ni***	***Pb***	***Zn***
*Pemba*	*Kigoma*	KIG 01	0.290	<0.001	114	58.6	85607	<0.001	289	59.0	<0.01	101
		KIG 02	0.490	<0.001	146	62.4	88382	<0.001	288	60.7	<0.01	104
		KIG 03	0.310	<0.001	111	63.1	89756	<0.001	283	63.4	<0.01	78.1
		KIG 04	0.270	<0.001	119	68.0	87269	<0.001	284	63.9	<0.01	80.4
		***Mean***	0.340	-	123	63.0	87754	-	286	61.8	-	90.9
		***SD***	0.101	-	16.0	3.9	1756	-	2.9	2.3	-	13.5
	*Musoma*	MSG 01	0.200	<0.001	65.0	27.5	34643	<0.001	1290	4.0	1.9	20.7
		MSG 02	0.190	<0.001	68.5	27.9	34534	<0.001	1312	4.7	2.8	23.1
		MSG 03	0.460	<0.001	98.3	58.9	34663	<0.001	1436	42.2	<0.01	68.3
		MSG 04	0.390	<0.001	94.7	58.9	35491	<0.001	1400	42.4	<0.01	28.1
		***Mean***	0.310	-	81.6	43.3	34833	-	1360	23.3	2.4	35.1
		***SD***	0.136	-	17.3	18.0	442	-	69.7	21.9	0.636	22.4
*Kichuguu*	*Katolo*	KTG 01	4.8	0.025	108	61.5	35878	0.039	861	51.3	7.4	45.5
		KTG 02	4.6	<0.001	98.5	62.3	57916	0.065	762	53.8	6.4	38.5
	*Rwamagasa*	RWG 01	14.8	0.092	97.3	79.7	55983	0.056	671	45.4	2.9	101
		RWG 02	19.7	0.220	287	169	68922	0.075	1325	128	3.9	112
	*Geita*	GTG 01	0.790	<0.001	103	46.4	33884	0.020	571	60.8	1.5	24.4
		GTG 02	3.0	0.044	41.5	67.4	43929	0.037	828	39.4	6.0	27.8
	*Kasamwa*	KSG 01	3.1	0.035	133	53.8	45600	0.039	1303	65.9	6.2	28.7
		KSG 02	4.5	0.035	68.8	58.7	51649	0.032	1243	43.4	5.8	30.7
	*Nyankumbu*	NYG 01	3.2	0.016	132	50.0	42919	0.015	1343	69.8	5.8	25.6
		NYG 02	5.3	<0.001	108	52.9	38765	0.041	529	55.9	7.0	26.8
	*Bukoli*	BKG 01	3.3	0.016	73.0	63.4	46401	0.022	761	83.1	9.4	41.8
		BKG 02	3.6	<0.001	99.9	51.1	45912	0.052	602	56.6	8.3	45.3
	*Chikobe*	CHG 01	5.0	<0.001	246	69.9	72204	0.072	2515	101	11.3	34.5
		CKG 02	5.9	0.016	216	61.4	56838	0.074	1251	113	8.7	29.2
		***Mean***	5.8	0.055	129	67.7	49771	0.046	1040	69.1	6.5	43.7
		***SD***	5.1	0.066	70.7	30.5	11501	0.020	522	27.3	2.6	27.6

As=arsenic; Cd=cadmium; Cr=chromium; Cu=copper; Fe=iron; Hg=mercury; Mn=manganese; Ni=nickel; Pb=lead; Zn=zinc.

**Table 4:** Estimated daily intake and daily dose of chemical elements of the soil eaten by pregnant women

***Chemical content***	***Kigoma Pemba***			***Musoma Pemba***			***Kichuguu soil***			MRLs* *(mg/kg/day)*
	**Mean conc. ( *mg/kg *)**	***Daily intake (mg/day)***	***Daily dose (mg/kg/day)***	**Mean conc. (mg/kg)**	***Daily intake (mg/day)***	***Daily dose (mg/kg/day)***	**Mean conc. ( *mg/kg *)**	***Daily intake (mg/day)***	***Daily dose (mg/kg/day)***	
*As*	0.340	0.021	0.0003	0.310	0.019	0.0002	5.8	0.36	0.0045	0.0003
*Cd*	*BDL*	*BDL*	*BDL*	*BDL*	*BDL*	*BDL*	0.036	0.002	0.00003	0.0005
*Cr*	122	7.6	0.095	81.6	5.1	0.064	129	8.1	0.100	0.0009^a^
*Cu*	63.0	3.9	0.049	43.3	2.7	0.034	67.7	4.2	0.053	0.010
*Fe*	*87754*	*5484*	*68.6*	34833	2177	27.2	49771	3111	38.9	Xx
*Hg*	*BDL*	*BDL*	*BDL*	*BDL*	*BDL*	*BDL*	0.046	0.003	0.00004	0.0003^b^
*Mn*	286	17.9	0.220	1360	85.0	1.1	1040	65.0	0.810	0.160^c^
*Ni*	61.8	3.9	0.048	23.3	1.4	0.018	69.1	4.3	0.054	*xx*
*Pb*	BDL	BDL	BDL	2.4	0.150	0.0019	6.5	0.410	0.005	xx
*Zn*	90.9	5.7	0.071	35.1	2.2	0.027	43.7	2.7	0.034	0.300

Mean body weight of 80 *kg* for an adult was used to estimate Daily Dose in women*, mg/kg/day*[46].

As=arsenic; Cd=cadmium; Cr=chromium; Cu=copper; Fe=iron; Hg=mercury; Mn=manganese; Ni=nickel; Pb=lead; Zn=zinc.

*Oral Minimal Risk Levels (MRLs) in *mg/kg/day* for chronic exposure to chemical element as established by ATSDR [45].

BDL refers to concentration below the method detection limit of the particular mineral element.

^a^Oral MRLs for chromium hexavalent.

^b^Oral MRLs for chronic toxicity for methyl mercury.

^c^Interim guidance value for manganese (*mg/kg/day*).

^xx^No oral MRLs have been derived for the specific chemical element.

The total chromium dose was found to be higher than the oral MRLs for chronic exposure for chromium hexavalent of 0.001 *mg/kg/day*[45] irrespective of the source of the sample. The same trend was observed for copper, where the *pemba* dose was estimated at 0.049 and 0.034 *mg/kg/day* for Kigoma and Musoma samples respectively and 0.053 *mg/kg/day* for *kichuguu* samples; all of these were above the the oral MRLs for intermediate exposure for copper of 0.010 *mg/kg/day*[21,45]. The dose for manganese was found to be higher in all the samples as compared to the interim guidance value for chronic exposure for manganese of 0.160 *mg/kg/day*[22,45], whereas the dose for zinc was found to be lower than the oral MRLs for chronic exposure of 0.300 *mg/kg/day*[23,45] in all of the samples analysed.

The daily intake for iron (5484, 2177 and 3111 *mg/day* for Kigoma and Musoma *pemba,* and *kichuguu* samples respectively) and are higher than the Tolerable Upper Intake Levels (UL) for iron intake of 45.0 *mg/day*, and above the Estimated Average Requirement (EAR) intake for iron of 22.0 *mg/day* for pregnant women aged from 19 to 50 years [25]. However, even though there have been reports that geophagy alleviates iron deficiency anemia of pregnancy [25], without adequate knowledge of a woman’s dietary intake of iron from food, water and supplements, it is unknown what contribution iron containing soil could make to achieve the EAR or exceed the UL.

## Discussion

In the present study, more than half of the respondents (59.7%) identified soil as substance ingested by pregnant women. This is consistent with a study conducted in Nairobi, Kenya, where 61.2% of the respondents reported soil as substance ingested [5]. We found that pregnant women purchase soil from local shops or eat soil taken from the ground. More pregnant women (45.6%) reported practicing geophagy in this study than previously reported in Tanzania, where the prevalence was estimated between 5.2% and 28.5% [6,9]. This reinforces suspicions of underreporting described in some studies [4,9].

Most of the pregnant women who ate soil started in the first trimester consistent with a previous study conducted in Kilimanjaro, Tanzania [9]. The authors also noted consumption of soil was used to treat morning sickness, nausea and vomiting [3,9,49]. In the current study, 31% of pregnant women who practice geophagy do so to prevent and/or stop morning sickness. However, beliefs vary as those not practicing geophagy did not associate soil eating with a reduction in morning sickness. The majority of all participants practicing geophagy did not believe that eating soil ensures a healthy pregnancy or prevents prolonged labor. This is in contrast with a large study reporting a positive belief towards preventing prolonged labor and ensuring a healthy pregnancy [49]. This may reflect regional differences in belief systems. Some participants mentioned that some women eat soil when they are not pregnant, and that it is also common in children and some men, and so geophagy is not restricted to pregnant women [10]. This indicates wider social and cultural considerations as an explanation for the practice.

The findings that pregnant women ingest soil up to three times per day are consistent with a study in Kenya [5]. We did not take blood samples or include questions regarding adverse symptoms associated with chemical element constituents of *pemba* and *kichuguu*. However, those women practicing geophagy potentially increased their exposure to chemical elements found in samples compared to those who did not consume soil. Soil from *kichuguu* in the Geita District is of particular concern because of potential soil contamination from arsenic among other chemical elements due to gold mining in the area [40,50]. The present study found total arsenic in the *kichuguu* samples at doses above the oral MRLs for chronic exposure of 0.0003 *mg/kg/day* for inorganic arsenic [30,45]. Establishing the bioavailability of soil constituents and determining - adverse effects calls for further study.

The levels of mercury found in the *kichuguu* soil in this study is consistent with other soil testing conducted in this area [50], suggesting that pregnant women who practice geophagy may be exposed to high levels of mercury. In addition, pregnant women are generally exposed to arsenic, chromium, copper, manganese and nickel at different levels depending on the type of soil eaten and the source obtained. As such, women who eat *kichuguu* from areas with minimal waste management practices, such as artisanal and small scale gold mining locations, are potentially at increased risk for exposure to chemical elements as compared to those who eat *pemba*, which generally comes from other locations.

Exposure to chemical elements has been associated with increased risks of a range of adverse neuro-cognitive developmental effects and increased neonatal and post-natal mortality, lowered birth weight, spontaneous abortion, increased number of still births and congenital malformations [19,20,28-35,45]. For instance, modest consumption of 50.0 *grams* of soil taken from an arsenic contaminated area per day is equivalent to intake of 0.370 *mg* of arsenic [1]. The presence of lead in some of the *pemba* and most of the *kichuguu* presents a risk of lead toxicity, which can severely damage the brain and kidneys in adults or children and may cause miscarriage and can ultimately cause death. [29,51]. Lead exposures either in utero, during infancy, or during childhood can result in delays or impairment of neurological development, neurobehavioral deficits, low birth weight and low gestational age, growth retardation, and delay maturation in girls [29,45,51]. Pregnant women who practice geophagy may expose themselves and their unborn babies to the risk of chemical elements some above the oral MRLs for either intermediate or chronic exposures. Risk to the fetus is even greater as the toxins concentrate from the mother to the fetus [34,35].

Even though copper, manganese, zinc and iron are essential elements for maintaining good health, high levels of each can have harmful effects [25]. According to the ATSDR, large doses of zinc and copper taken by mouth can cause stomach cramps, nausea, vomiting and even death [21,23,45]. Manganese is an essential nutrient involved in bone formation and carbohydrate metabolism but high intake levels of manganese can result in “manganism” [22]. This disease, usually characterized as an occupational hazard for people who inhale manganese dust, results in neurological effects similar to Parkinson’s disease [25]. The Institute of Medicine cautions against taking manganese supplements in individuals who consume plant products high in manganese [25]. The recommended average intake (AI) during pregnancy for manganese is *2.0 mg/day* and tolerable upper intake level is 11.0 *mg/day*[25]. Estimates of samples of Musoma *pemba,* Kigoma *pemba,* and *kichuguu* consumed daily by pregnant women contained 17.9, 85.0 and 65.0 *mg/day* respectively (Table 4). These levels exceed recommendations. In addition, higher than recommended amounts of zinc consumed for prolonged periods can cause anemia and decrease levels of high-density lipoprotein cholesterol [23]. The amount of zinc found in the soil eaten calls for further monitoring due to potential multiple sources of zinc from food sources.

Overall evidence indicates that those working in public health and health care delivery should discourage geophagy, particularly when the soil sources are from settings such as gold mining where environmental soil contamination is likely. However, because of the complexity underlying soil eating behaviors and a lack of consensus about why women undertake this practice, elimination of geophagy will be challenging. Thus, a range of options could be applied to artisanal mining settings including improved waste management practices, establishing educational programs for health workers and mothers, introducing a surveillance system that focus on exposure and/or outcomes, providing safer alternatives to soil for eating, or even ensuring that the soil eaten by pregnant women is from a safer source free of contaminants.

## Conclusions

The findings revealed a higher prevalence of geophagy among pregnant women surveyed in Geita District, a gold mining region, than reported across Tanzania, and in many other studies. Current practices may be explained by women’s need to manage nausea associated with pregnancy. However, the health beliefs and cultural meaning given to the practice requires further exploration. Importantly, potentially harmful exposure to chemical elements in the soil contaminated by mining varies depending upon frequency and amount consumed, but we have shown that most samples exceeded established safety levels. Thus, in artisanal mining settings, culturally appropriate and sensitive policies and programs should be developed that directly address a reduction of exposure to contaminants from geophagy.

## Endnote

^*a*^*mg/kg/day* = *Concentration of the mineral element in mg/kg x Soil Ingestion rate in kg/day per mean adult body weight in kg.*

## Competing interests

The authors declare that they have no competing interests, and neither the principal investigators nor the co-investigators have actual or potential conflict of interest.

## Authors’ contribution

ECN contributed in designing the study, developing the survey, supervising the data collection, analyzing the data, overseeing laboratory testing of soil samples, and drafting and writing of the manuscript. MJ developed the survey, supervised the data collection, analysed the data, coded the open ended question, and interpreted the results. SSP contributed to the development of the study design and the survey, analysed the data, interpreted the results, and writing the manuscript. DSKT contributed to the study design and development of the survey, the interpretation of the findings, as well as the writing of the manuscript. CM contributed to the interpretation of results and reviewing the manuscript. All authors read and approved the final manuscript.

## Pre-publication history

The pre-publication history for this paper can be accessed here:

http://www.biomedcentral.com/1471-2393/14/144/prepub
